# Regulation of cargo transfer between ESCRT-0 and ESCRT-I complexes by flotillin-1 during endosomal sorting of ubiquitinated cargo

**DOI:** 10.1038/oncsis.2017.47

**Published:** 2017-06-05

**Authors:** M Meister, S Bänfer, U Gärtner, J Koskimies, M Amaddii, R Jacob, R Tikkanen

**Affiliations:** 1 Institute of Biochemistry, Medical Faculty, Justus-Liebig University of Giessen, Giessen, Germany; 2 Department of Cell Biology and Cell Pathology, Philipps University of Marburg, Marburg, Germany; 3 Institute of Anatomy and Cell Biology, Medical Faculty, Justus-Liebig University of Giessen, Giessen, Germany

## Abstract

Ubiquitin-dependent sorting of membrane proteins in endosomes directs them to lysosomal degradation. In the case of receptors such as the epidermal growth factor receptor (EGFR), lysosomal degradation is important for the regulation of downstream signalling. Ubiquitinated proteins are recognised in endosomes by the endosomal sorting complexes required for transport (ESCRT) complexes, which sequentially interact with the ubiquitinated cargo. Although the role of each ESCRT complex in sorting is well established, it is not clear how the cargo is passed on from one ESCRT to the next. We here show that flotillin-1 is required for EGFR degradation, and that it interacts with the subunits of ESCRT-0 and -I complexes (hepatocyte growth factor-regulated tyrosine kinase substrate (Hrs) and Tsg101). Flotillin-1 is required for cargo recognition and sorting by ESCRT-0/Hrs and for its interaction with Tsg101. In addition, flotillin-1 is also required for the sorting of human immunodeficiency virus 1 Gag polyprotein, which mimics ESCRT-0 complex during viral assembly. We propose that flotillin-1 functions in cargo transfer between ESCRT-0 and -I complexes.

## Introduction

Signalling by receptors such as the epidermal growth factor receptor (EGFR) is downregulated by degradation of the receptor in lysosomes, which requires the receptor to be ubiquitin modified. Ubiquitinated cargo destined for lysosomal degradation is recognised and sorted by the endosomal sorting complexes required for transport (ESCRTs; reviewed in Henne *et al.*^1^). ESCRTs sequentially bind to the ubiquitinated cargo in endosomes and sequester it into intralumenal vesicles, thereby generating multivesicular bodies that deliver the cargo to lysosomes.^2, 3^ The initial recognition of ubiquitinated cargo and recruitment of ESCRT-I is conducted by the ESCRT-0 complex, which contains two subunits, hepatocyte growth factor-regulated tyrosine kinase substrate (Hrs) and signal-transducing adaptor molecule 1/2 (STAM 1/2). Hrs contains a VHS (Vps27, Hrs and STAM) domain, a lipid-binding FYVE domain,^4, 5, 6^ a UIM (ubiquitin-interacting motif) domain, a coiled-coil domain and a clathrin-binding domain at the C-terminal end.^7, 8, 9^ ESCRT-I is recruited by the binding of its subunit Tsg101 to a P(S/T)AP motif in Hrs, located between the UIM and the coiled-coil domain.^10, 11, 12, 13^ ESCRTs not only have a role during receptor sorting but also many viruses, such as the human immunodeficiency virus 1 (HIV-1), hijack the ESCRT-mediated sorting machinery during particle assembly and viral budding.^14^

Flotillin-1 and flotillin-2 are ubiquitously expressed, membrane raft-associated proteins that have been connected to endosomal trafficking of the β-secretase BACE1^15^ and of bacterial toxins.^16, 17^ Despite their high degree of conservation, the molecular function of flotillins has remained somewhat obscure. A number of studies have implicated that flotillins may function in membrane trafficking, signalling and cell adhesion by means of their high propensity to oligomerise, and may thus function as scaffolders in various biological processes (for a review, see Meister and Tikkanen^18^). However, flotillins can also directly interact with sorting signals in cargo such as BACE1,^15^ but their exact role in endosomal trafficking has not been dissected.

Although the role of ESCRTs during degradative sorting of ubiquitinated cargo is well known, the details regarding how the cargo is passed on from one ESCRT to the next during the sorting are not clear. We here show that flotillin-1, which we have previously demonstrated to regulate EGFR signalling but not EGFR uptake from the plasma membrane,^19^ is involved in the recognition and sorting of ubiquitinated cargo by ESCRT-0 and -I.

## Results

### EGFR degradation is attenuated in flotillin-depleted cells

**Figure 1:**
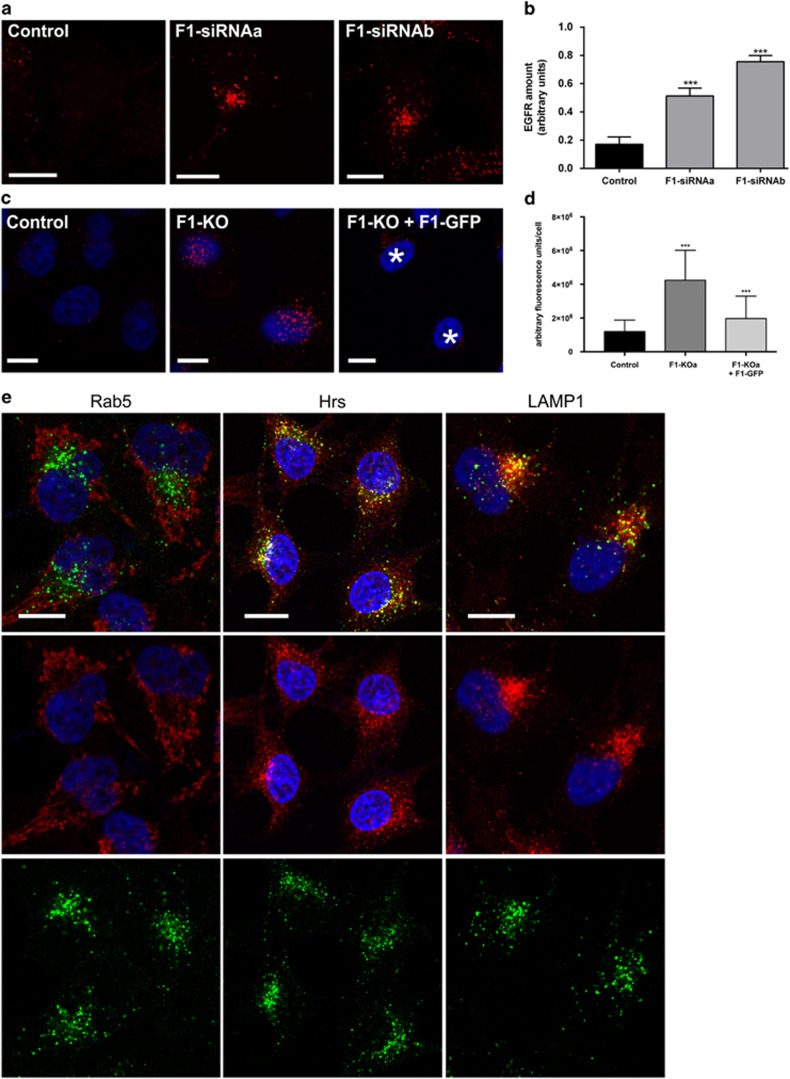
EGFR degradation is impaired in the absence of flotillin-1.(**a**, **b**) Control siRNA, flotillin-1 (F1-siRNAa and -b) and (**c**, **d**) control HeLa or flotillin-1-knockout cells (transfected with flotillin-1-EGFP or not) were starved overnight and stimulated with 100 ng/ml EGF for 60 min, fixed with methanol and immunostained for EGFR. (**b**) The amount of undegraded EGFR in flotillin-1-knockdown cells was quantified as fluorescence intensity in arbitrary units using the ImageJ Software. A significant increase of EGFR signal was observed in flotillin-knockdown cells as compared with the control. (**d**) EGFR amount after 60 min was quantified as in **c**, and the data show a rescue of EGFR degradation upon flotillin-1-EGFP expression (cells marked with *, images for GFP in Supplementary Figure S1d). The data in **c**, **d** are shown as mean±s.d. Cells from three independent experiments were evaluated (control siRNA cells: *n*=136, F1-siRNAa: *n*=135 and F1-siRNAb: *n*=149; control HeLa: *n*=358, flotillin-1 KO: *n*=181, rescue cells: *n*=252). Statistical analysis was performed with one-way analysis of variance (ANOVA) and Bonferroni post-test in comparison with the control. ***P<0.001. Scale bar in (**a**, **c**, **e**): 10 μm. (**e**) Flotillin-1-knockdown cells (F1-siRNAb) were treated as in **a** and immunostained for EGFR (green) and Rab5, Hrs or LAMP1 (red).

Our earlier findings have shown that although flotillin-1 affects EGFR signalling, it is not involved in the early uptake of EGFR from the plasma membrane upon EGF stimulation.^19^ However, upon prolonged EGF stimulation for 60 min, an accumulation of EGFR was observed in intracellular, perinuclear structures in flotillin-1- (Figure 1a) and flotillin-2- (Supplementary Figure S1a) small interfering RNA (siRNA) knockdown HeLa cells, whereas in control siRNA cells, only a very low signal for EGFR was detected (Figure 1a). Quantification showed a significant increase in non-degraded EGFR in flotillin-1-knockdown cells (Figure 1b). Flotillin depletion phenocopied a knockdown of Golgi-localised, γ-ear containing, ADP-ribosylation factor binding protein 3 (GGA3) (Supplementary Figure S1a), which has been shown to impair EGFR degradation.^20^ Knockdown efficiencies are depicted in Supplementary Figure S1b; please note that flotillin-2 depletion also severely impairs flotillin-1 expression, suggesting that the observed defect is more because of flotillin-1 ablation.

To exclude that the reduced degradation is an unspecific effect of the siRNAs used, we generated flotillin-1-knockout HeLa cells using the CRISPR/Cas9 method,^21^ which resulted in complete absence of flotillin-1 expression (Supplementary Figure S1c). The EGFR degradation defect was also visible in the flotillin-1-knockout cells, and it could be significantly rescued by expression of flotillin-1-GFP (Figures 1c and d and Supplementary Figure S1d shows GFP expression).

As EGFR appeared to accumulate in a perinuclear compartment, we performed colocalization studies of EGFR and endosomal markers in flotillin-1-knockdown cells stimulated with EGF for 60 min (Figure 1e). Whereas the early endosome marker Rab5 showed a very limited colocalization with the EGFR (Pearson’s coefficient *R*=0.134±0.060), a higher degree of colocalization was observed with the more terminal markers Hrs (*R*=0.442±0.093) and LAMP1 (*R*=0.503±0.103; values based on the assessment of three independent experiments, with more than 20 cells per condition per experiment). These data suggest that EGFR is trapped in late endosomes in the absence of flotillin-1.

### Retention of ubiquitinated cargo at late endosomes (LE) upon flotillin knockdown

**Figure 2:**
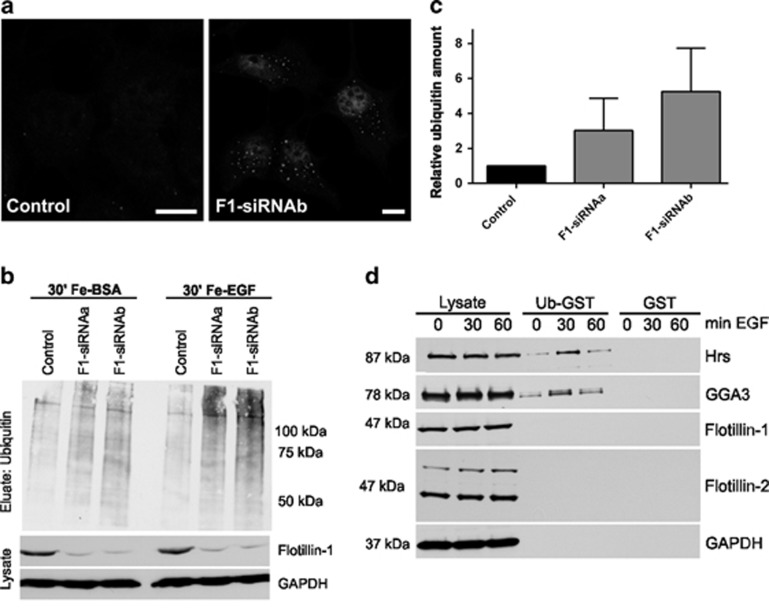
Increased amount of ubiquitinated cargo in endosomes of EGF-stimulated flotillin-1-knockdown cells.(**a**) Control siRNA and flotillin-1 siRNA-transfected cells were stimulated with 100 ng/ml EGF for 30 min, fixed and immunostained for ubiquitin. An enhanced ubiquitin staining was observed in punctate perinuclear structures in flotillin-1-knockdown cells. Scale bar: 10 μm. (**b**) Endosomes were isolated with magnetic columns from control siRNA and flotillin-1-depleted cells treated with Ferrofluid-coupled BSA or Ferro-EGF for 30 min. Samples from homogenate and eluate were analysed for ubiquitin by western blot. (**c**) Increased ubiquitin signal in endosomes of flotillin-1-knockdown cells. Densitometric analysis of ubiquitin signals shown in **b** from three independent experiments±s.d. Data were normalised to GAPDH in homogenates. (**d**) GST pulldown with ubiquitin-GST or GST from HeLa cells treated for 0, 30 or 60 min with EGF shows an increased binding of Hrs and GGA3 to ubiquitin upon EGF stimulation, whereas flotillins do not bind to ubiquitin-GST. Ubiquitin interactors were detected by western blot using specific antibodies, GAPDH was used as an input control.

Flotillin-1 knockdown resulted in increased staining of ubiquitin in vesicular structures after 30 min EGF stimulation as compared with control cells (Figure 2a). To biochemically quantify endosomal ubiquitin, we incubated control and flotillin-1-knockdown cells with Ferrofluid-coupled EGF (Fe-EGF) or bovine serum albumin (BSA) for 30 min and then magnetically isolated endosomes. A tendency to an increased amount of ubiquitinated proteins was observed in flotillin-1-knockdown cells (Figures 2b and c). These data suggest that ubiquitinated cargo accumulates in endosomes upon flotillin knockdown, similarly to Hrs-overexpressing cells,^22^ and that flotillin-1 might function as an LE sorting factor for ubiquitinated cargo. The activity of Fe-EGF was demonstrated by measuring the phosphorylation of extracellular signal-regulated kinase after 5 min of Fe-EGF stimulation (Supplementary Figure S1e). Consistent with our previous data,^19^ Fe-EGF resulted in diminished extracellular signal-regulated kinase activation in flotillin-1-knockdown cells. Many LE sorting factors, including Hrs and GGA3, can directly bind to ubiquitin (Figure 2d and Supplementary Figure S1f), whereas flotillins showed no ubiquitin binding in a GST-pulldown assay (Figure 2d).

### Enlargement of endosomes upon flotillin-1 depletion

**Figure 3:**
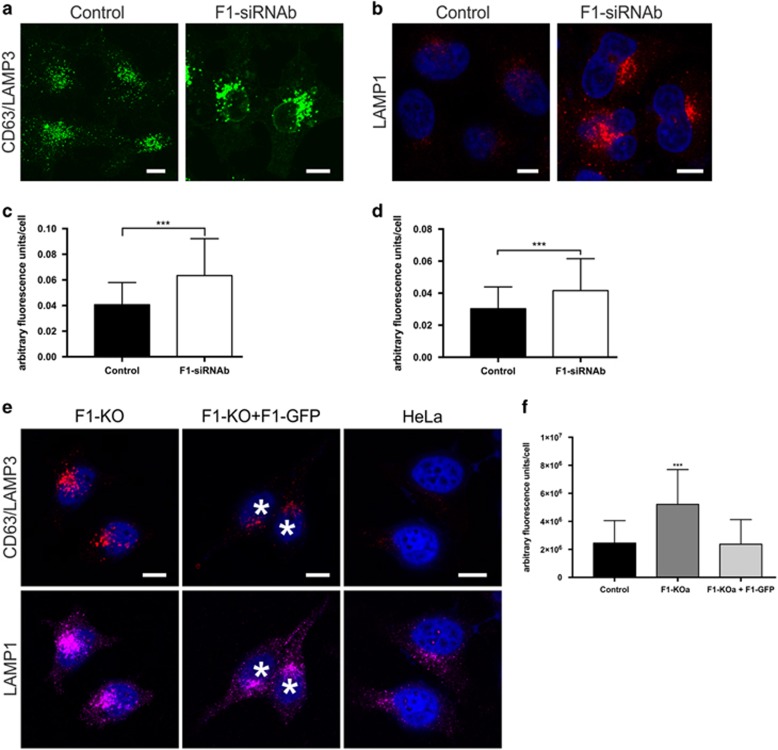
Changes in endosomal and lysosomal morphology upon flotillin-1 knockdown. Immunofluorescent labelling for (**a**) LAMP3 or (**b**) LAMP1 in control siRNA and flotillin-1-knockdown HeLa cells. Quantification of the stainings for (**c**) LAMP3 and (**d**) LAMP1 show a significantly increased staining intensity for both endosomal markers in flotillin-1-knockdown cells. (**e**) Flotillin-1-knockout cells (F1-KO) show a similar phenotype with intense LAMP3 and LAMP1 staining that can be rescued upon expression of flotillin-1-EGFP (cells marked with *, images for GFP in Supplementary Figure S1g). (**f**) Quantification of the LAMP3 staining in **e**. The data in **c**, **d**, **f** are shown as mean±s.d. Cells from three independent experiments were evaluated (control siRNA cells: *n*=136, F1-siRNAa: *n*=135 and F1-siRNAb: *n*=149; control Hela: *n*=149, flotillin-1 KO: *n*=211, rescue cells: *n*=147). Statistical analysis in **c**, **d** was performed with Student’s *t*-test (two-tailed, unpaired), and in **f** with one-way analysis of variance (ANOVA) and Bonferroni post-test, in comparison with the control. ***P<0.001. Scale bar in **a**, **b**, **e**: 10 μm.

**Figure 4:**
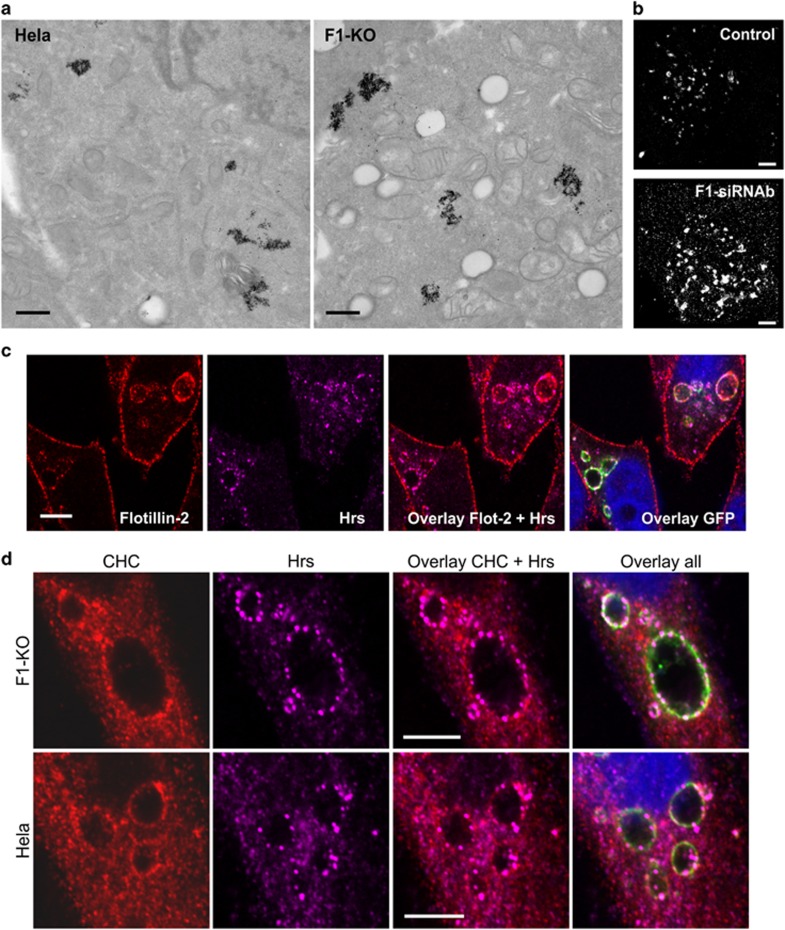
Endosomal morphology is altered in flotillin-1-knockdown/knockout cells, but endosomal domains persist.(**a**) Control and flotillin-1-knockout cells were chased for 2 h with Fe-EGF, fixed and analysed by transmission electron microscopy. Note the enlarged endosomes in flotillin-1-knockdown cells. Scale bar: 500 nm. (**b**) Control siRNA and flotillin-1-knockdown cells were stimulated with EGF for 30 min and labelled with an anti-mouse LAMP3 antibody detected with a secondary Alexa Fluor 647-coupled antibody. The specimens were analysed with Leica SR GSD 3D microscope. Scale bar: 2 μm. (**c**) Rab5-Q79L-GFP-expressing HeLa cells were immunostained for endogenous flotillin-2 and Hrs, which show a limited colocalization in the enlarged endosomes. Scale bar: 10 μm. (**d**) Rab5-Q79L-GFP-expressing HeLa and flotillin-1-knockout cells were immunostained for endogenous CHC and Hrs, which are localised in endosomal domains in both cell types and show some colocalization. Scale bar: 10 μm.

Immunofluorescent staining of control and flotillin-1-knockdown cells with LE/lysosomal markers (LAMP3 and LAMP1) showed an altered morphology and a significant increase in the staining of these structures upon flotillin-1 depletion (Figures 3a–d), suggesting an enlargement of endosomes upon loss of flotillin-1. The same phenotype was observed in flotillin-knockout cells, and again it could be significantly rescued upon re-expression of flotillin-1 (Figures 3e and f and Supplementary Figure S1g). Electron microscopy of cells chased for 2 h with Fe-EGF revealed more translucent endosomal structures in flotillin-1-knockout cells, which looked enlarged as compared with the control cells (Figure 4a, larger images in Supplementary Figures S2a and b). However, Fe-EGF did not accumulate in these structures, suggesting that they do not represent lysosomes. To verify these data, ground-state depletion (GSD) super-resolution microscopy was applied for this purpose. This method provides a resolution of about <30 nm,^23^ allowing the evaluation of endosomal size in sections covering the whole cell depth. GSD microscopy after staining for LAMP3 revealed enlarged late endosomes in flotillin-1 knockdown as compared with control siRNA cells (Figure 4b). Owing to the large heterogeneity in the endosomal size, these data were not assessed quantitatively. Three-dimensional images of the GSD stainings are depicted in Supplementary Figures S2c and d. These data are again consistent with a putative role for flotillin-1 as an endosomal sorting factor, as depletion of, for example, Hrs also results in enlargement of endosomes.^4, 24^

### Flotillins and Hrs colocalize in endosomes

To study if flotillins colocalize with endosomal sorting complexes, we performed double stainings for flotillin-2 and Hrs. Since the staining intensities for these endogenous proteins were not sufficient for GSD microscopy, we used overexpression of the Q79L mutant of the GTPase Rab5 (Rab5-Q79L-GFP) that induces an enlargement of endosomes by increasing the fusion rate. This method has previously been used to study endosomal sorting domains and Hrs function.^8, 25^ We observed a punctate colocalization of endogenous Hrs and flotillin-2 in the enlarged endosomes (Figure 4c). Some of the Hrs containing domains were positive for clathrin heavy chain (CHC; Figure 4d), and these domains also persisted in flotillin-1-knockout cells, implicating that the function of flotillin-1 in endosomal sorting is not based on its propensity to function as a domain scaffolder.^18^

### Flotillins are required for Hrs ubiquitin binding

**Figure 5:**
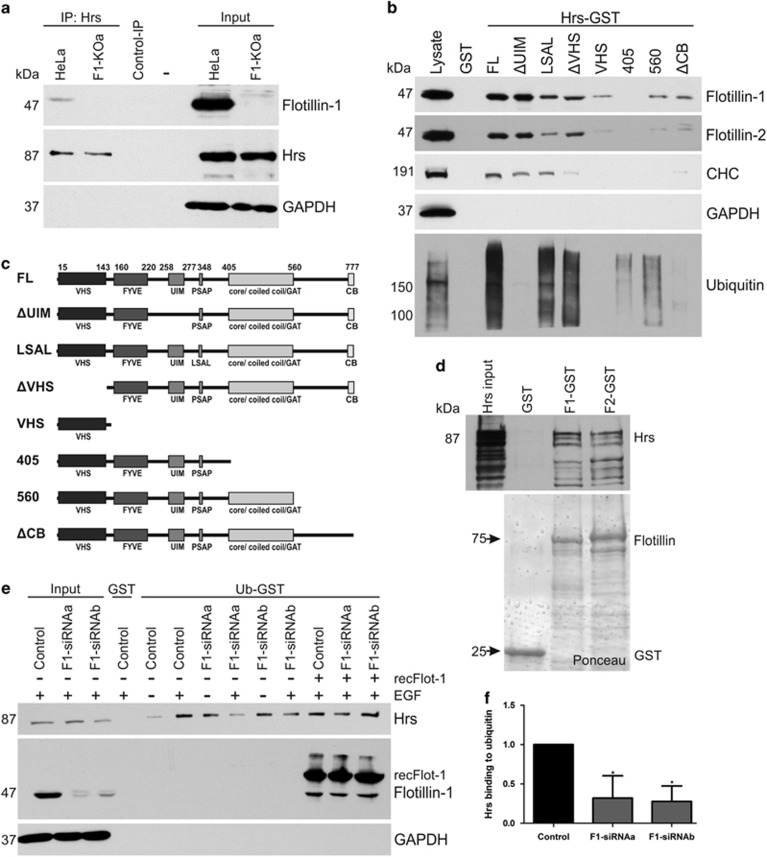
Flotillins directly interact with Hrs, and flotillin-1 is required for Hrs interaction with ubiquitin.(**a**) Co-immunoprecipitation of endogenous flotillin-1 with Hrs from HeLa cells stimulated with EGF for 30 min. Flotillin-1-knockout cells were used as a control to identify flotillin-1 bands. Control immunoprecipitation from HeLa cells with an isotype-matched antibody does not show any co-precipitation. (**b**) Purified Hrs-GST proteins were used to pull down flotillins from HeLa lysates for the mapping of the interaction sites of flotillins in Hrs. Flotillin interaction with Hrs is not dependent on the UIM domain in Hrs, whereas mutation of the PSAP motif into LSAL or deletion of the VHS domain partially abrogates flotillin interaction. VHS domain alone shows only a minor binding to flotillins. Deletion of the C-terminal region including the core/coiled-coil/GAT domains abolishes Hrs interaction with flotillins, and deletion of the C-terminal region beyond these domains results in decreased binding. Deletion of the clathrin-binding motif (CB) in Hrs C terminus also abrogates flotillin binding. (**c**) Structure of the constructs used in **a**. (**d**) Hrs and flotillins directly interact. GST pulldown was performed with recombinant, purified proteins. GST-tag in Hrs was removed before pulldown using thrombin digestion. (**e**) Interaction of endogenous Hrs with ubiquitin in an ubiquitin-GST-pulldown assay is reduced in EGF-stimulated (30 min) flotillin-1-knockdown cells, but can be rescued by the addition of purified recombinant flotillin-1. The pulldown samples were analysed by western blot using Hrs, flotillin-1 and GAPDH antibodies. (**f**) Quantification of Hrs binding to ubiquitin-GST upon 30 min EGF. Statistical analysis was performed using one-way analysis of variance (ANOVA) with Bonferroni post-test. The values shown represent mean±s.d. from three independent experiments. *P<0.05.

Our data showed that flotillins do not directly bind ubiquitin, thus they are more likely to exert their function by binding to the endosomal sorting complexes and/or cargo. Consistently, endogenous flotillin-1 co-immunoprecipitated with Hrs from HeLa cells treated for 30 min with EGF (Figure 5a). Furthermore, flotillins associated with Hrs and Tsg101 in GST-pulldown experiments (Supplementary Figure S3a). Further dissection of the flotillin-1 binding site in Hrs (Figures 5b and c) showed that deletion of the UIM domain of Hrs did not impair flotillin interaction, whereas mutation of the PSAP motif into LSAL or deletion of the VHS domain moderately reduced flotillin interaction with Hrs, but the VHS domain alone poorly associated with flotillins. However, deletion of the C-terminal Hrs region containing the GAT domain completely prevented flotillin binding, whereas deletion beyond GAT domain and mutation of the clathrin interaction motif (clathrin-binding domain) resulted in largely reduced interaction with flotillin-1 (Figures 5b and c and Supplementary Figure S3b). However, Hrs mutants with impaired flotillin binding retained their ability to interact with ubiquitin in a pulldown assay (Supplementary Figure S3c).

Flotillins directly interact with Hrs, as shown with a pulldown using purified recombinant proteins (Figure 5d). Interestingly, flotillin-1 depletion significantly impaired the ability of Hrs from EGF-stimulated cells to bind ubiquitin in a GST-pulldown assay (Figures 5e and f and Supplementary Figure S3d). Addition of purified recombinant flotillin-1 in the pulldown resulted in a partial rescue of Hrs ubiquitin binding (Figure 5e), but the degree of rescue was highly variable between experiments.

**Figure 6:**
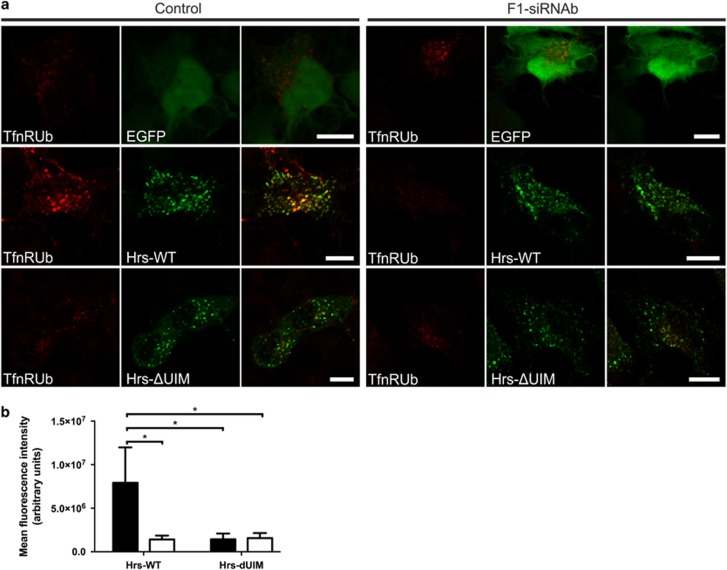
Flotillin-1 is necessary for the sorting of ubiquitinated cargo by ESCRT-0. (**a**) Control siRNA and flotillin-1-knockdown HeLa were transfected with TfnR-Ub chimera and Hrs-WT-GFP, ΔUIM-Hrs-GFP or EGFP and starved overnight. The cells were treated with Tfn-546 for 15 min, washed and chased for 60 min to allow for Tfn uptake. In control cells, Tfn accumulates in Hrs-WT-EGFP-positive structures, whereas in flotillin-1-knockdown cells, no accumulation is seen, indicating impaired Hrs–cargo binding. Expression of ΔUIM-Hrs does not result in Tfn accumulation, consistent with the inability of this mutant to bind ubiquitin. Scale bar: 10 μm. (**b**) Flotillin-1-knockdown cells accumulate significantly less Tfn after Hrs-WT-EGFP transfection. The mean fluorescence intensity of the Tfn-546 signal from control siRNA and flotillin-1-knockdown cells transfected with Hrs-WT-GFP, ΔUIM-Hrs-GFP and TfnR-Ub after 15 min uptake and 60 min chase was measured. Quantification of Tfn signals as arbitrary units was carried out with the ImageJ software. Bars show the mean±s.d. from three independent experiments (>90 cells per condition). Two-way analysis of variance (ANOVA) with Bonferroni post-test. *P<0.05.

The effect of flotillin knockdown on Hrs-mediated cargo sorting was next tested in a sorting assay using a transferrin receptor-ubiquitin chimera that binds transferrin but is sorted in an Hrs-dependent manner and not recycled.^7^ It has been shown that overexpression of Hrs impairs endosomal sorting of ubiquitinated cargo by inhibition of intralumenal vesicle formation.^22, 26^ Thus, in Hrs-overexpressing cells, the transferrin receptor-ubiquitin fusion is bound by Hrs and accumulates in endosomes, which can be followed using fluorescently labelled transferrin.^7^ Strong endosomal accumulation of transferrin was observed in control cells expressing WT Hrs (Figure 6a, expression of transfected proteins in Supplementary Figures S4a and b). In cells expressing an Hrs mutant in which the UIM domain has been deleted, no transferrin accumulation was observed due to recycling and release in culture medium, consistent with the data of Raiborg *et al.*^7^ However, in WT Hrs-overexpressing flotillin-1-knockdown cells, no accumulation of Tfn was observed (Figure 6a, quantified in Figure 6b), demonstrating that Hrs is not capable of sorting ubiquitinated cargo in the absence of flotillin-1.

### Hrs expression does not rescue EGFR degradation

To further study the cooperation of flotillin-1 and Hrs in cargo sorting, the ability of Hrs overexpression to rescue EGFR degradation was tested in flotillin-1-knockout cells. However, overexpression of Hrs-EGFP or the mutant dCB lacking the clathrin-binding domain did not ameliorate the EGFR degradation defect in the absence of flotillin-1 (Supplementary Figure S4c). As reported previously, overexpression of these Hrs variants resulted in an impairment of EGFR degradation in HeLa cells. Thus, Hrs and flotillin-1 are likely to be tightly functionally associated during ubiquitin-bound cargo sorting.

### Flotillin-1 depletion impairs cargo delivery from ESCRT-0

**Figure 7:**
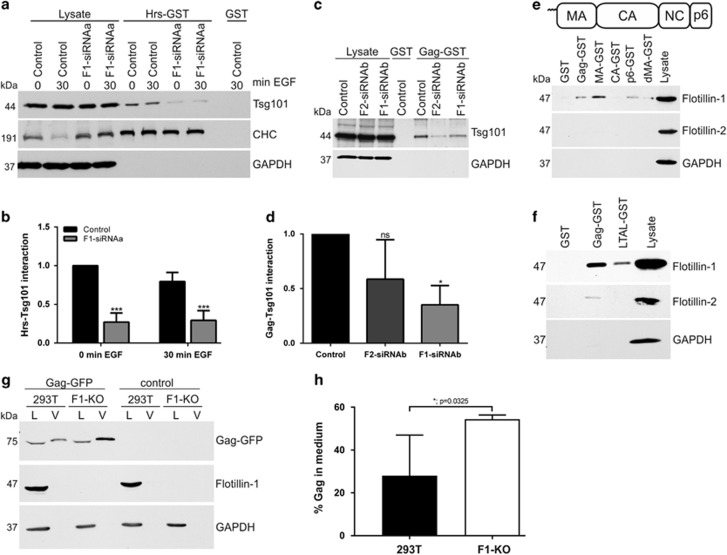
Flotillin-1 is required for the binding of Hrs and HIV-1 Gag to Tsg101, and for endosomal localisation of Gag-EGFP.(**a**) Binding of Tsg101 and CHC from starved and EGF (100 ng/ml) stimulated cells transfected with control siRNA or flotillin-1 siRNA to Hrs-GST was tested by western blot. (**b**) Flotillin-1 depletion significantly impairs Hrs-GST binding to Tsg101. (**c**) Binding of endogenous Tsg101 to HIV-Gag-GST was determined in flotillin-1 and flotillin-2 knockdown HeLa cells. (**d**) Significantly less Tsg101 was bound to Gag-GST from flotillin-depleted cells. (**e**) Binding of endogenous flotillin-1 and flotillin-2 to full-length Gag and its subdomains (MA, CA, p6 and dMA-Gag) expressed as GST fusion proteins was analysed in HeLa cell lysates. Uppermost part shows the structural domain organisation of Gag. (**f**) Binding of flotillin-1 to Gag is reduced by ablation of the PTAP motif. (**g**) VLPs from Gag-EGFP-transfected 293T control and flotillin-1-knockout cells were prepared, and analysed by western blot. L: lysate; V: VLP. (**h**) The amount of Gag in the cell lysates and in the medium was determined by scanning densitometry. The percentage of released Gag was calculated taking into account the total volumes of each fraction, and the data are presented as % total Gag released in the medium. Densitometric quantification and statistical analysis in **b**, **d**, **g** were performed with Quantity One (Bio-Rad) or GraphPad Software. Statistical analysis was carried out with two-way analysis of variance (ANOVA) with Bonferroni post-test. Data in **f** were analysed with Student’s *t*-test (two-tailed, unpaired). Data are shown as the mean±s.d. of three to four independent experiments. *P<0.05; ***P>0.001.

During sorting, the cargo delivery from ESCRT-0 to ESCRT-I is mediated by the interaction of Hrs and Tsg101.^12^ Our data show that flotillin-1 interacts with both Hrs and Tsg101, which might implicate a role in cargo transfer between ESCRT-0 and -I complexes. In control cells, Tsg101 from unstimulated and 30 min EGF-stimulated cells bound to Hrs-GST, whereas this binding was significantly reduced in flotillin-1-knockdown cells (Figures 7a and b and Supplementary Figure S5a). However, binding of CHC to Hrs was not impaired in flotillin-1-knockdown cells (Supplementary Figure S5b).

To test if cargo delivery to ESCRT-I is generally dependent on flotillin-1, we made use of HIV-1 that uses ESCRT-I, but not ESCRT-0, complex for the budding of viral particles. During HIV-1 assembly and budding, the polyprotein Gag mimics ESCRT-0/cargo complex and binds to Tsg101,^13^ which then aids the sorting of viral proteins into forming viral buds. Indeed, the binding of Tsg101 from flotillin-1-knockdown cell lysates to Gag-GST was reduced to ~35% of control cells (Figures 7c and d and Supplementary Figure S5c). Analogously to its interactions with Hrs/ESCRT-0, flotillin-1 also interacted with Gag-GST, especially with the Matrix (MA) and p6 parts of Gag (Figure 7e and Supplementary Figure S5d). Deletion of the MA part resulted in reduction of the interaction to about the level of the p6 part. To test if the PTAP motif contributes to flotillin binding, we mutated it to LTAL in full-length Gag-GST. Consistently, the LTAL-Gag exhibited a severely reduced flotillin-1 binding (Figure 7f and Supplementary Figure S5d). The release of virus-like particles (VLPs) was significantly higher from Gag-EGFP-transfected flotillin-1-knockout 293T cells than from control cells (Figures 7g and h), suggesting that flotillin-1 indeed is functionally associated with HIV assembly. Highly similar data were obtained in flotillin-1-knockdown HeLa cells (Supplementary Figure S5e). These findings suggest that flotillin-1 is also involved in the ESCRT/ubiquitin-mediated sorting of HIV-1 proteins during viral assembly and may have a general role in endosomal ubiquitin-mediated cargo sorting.

## Discussion

Our study reveals a novel role for the membrane raft-associated flotillin-1 as a regulator of ubiquitin- and ESCRT-mediated endosomal sorting. Although flotillin-1 has been suggested to be involved in clathrin-independent endocytosis,^27^ it does not participate in the uptake of EGFR from the plasma membrane.^19^ However, we here show that flotillin-1 depletion impairs EGFR degradation due to an endosomal sorting defect. We have previously shown that flotillins are important for the endosomal sorting of the β-secretase BACE1, which accumulates in endosomes upon flotillin depletion.^15^ Sorting of BACE1 to lysosomes, similarly to EGFR, is ubiquitin-dependent and involves the GGA3 adaptor protein,^28, 29^ whereas the role of ESCRTs has, to our knowledge, not been addressed. Nevertheless, endosomal sorting of both BACE1 and EGFR evidently requires flotillin-1, suggesting that flotillin-1 may have a general role in the degradative sorting of membrane proteins. However, flotillins do not directly bind ubiquitin, nor have we been able to demonstrate ubiquitination of flotillin-1 (our unpublished data).

We here show that depletion of flotillin-1 by means of siRNA or genetic ablation impairs but does not completely prevent EGFR degradation. Importantly, knockdown of Hrs results in only a modest defect in EGFR degradation and in enlargement of endosomes,^4, 30^ similarly to flotillin-1 depletion, as shown here. On the other hand, the absence of Tsg101 results in a more severe abrogation of EGFR degradation and formation of tubular endosomes,^22, 24^ implicating that flotillin-1 may act more at the level of ESCRT-0. Genetic ablation of either Hrs or Tsg101 expression in the mouse is embryonic lethal,^31, 32^ whereas flotillin-1- and flotillin-2-knockdown mice are viable and without any evident developmental defects.^33, 34^ Furthermore, flotillin-like proteins are not present in some organisms such as *Saccharomycescerevisiae* or *Caenorhabditiselegans* that use ESCRT-mediated endosomal cargo sorting. However, alternative routes that may be flotillin-independent are likely to exist, such as the GGA3-dependent pathway.^20^ Furthermore, it has been shown that even lipids, such as lysobisphosphatidic acid and ceramides, may in some cases drive intralumenal vesicle formation (for a review, see Babst^35^). Thus, ubiquitin-mediated sorting in eukaryotic and even multicellular organisms appears to be flexible and possible in the absence of flotillins, although there are no direct data on the efficiency of cargo sorting in flotillin-knockout animals.

Intriguingly, flotillin-1 also mediates HIV-1–Gag interaction with Tsg101, which is necessary for the sorting of viral proteins into the forming bud. HIV budding has been shown to take place in raft-like microdomains ('barges'),^36, 37^ but the budding can take place either in endosomes (in monocytic cells) or at the plasma membrane (in T cells) and may involve Gag endocytosis.^38, 39, 40^ However, irrespective of the actual site of HIV budding, the same machinery appears to be involved. This is also consistent with the localisation of flotillins in endosomes and at the plasma membrane, depending on the cell type.^41^ HIV budding takes place independently of Hrs/ESCRT-0, as HIV-Gag becomes ubiquitinated and mimics Hrs–cargo complex, interacting with Tsg101 by means of the late domain PTAP motif in Gag.^13^ Interestingly, the interaction of Gag with flotillin-1 is partly mediated by the MA domain that also mediates Gag membrane association, but the p6 fragment/PTAP motif also seems to contribute to the interaction. This is very similar to flotillin-1 interaction with Hrs, which also appears to be modulated by the Hrs PSAP motif and also requires other determinants in Hrs. As with Hrs–Tsg101 interaction, binding of Tsg101 to Gag is diminished in the absence of flotillin-1, supporting the conclusion that flotillin-1 facilitates cargo transfer from ESCRT-0 (or Gag) to ESCRT-I. However, flotillin-1 depletion appears to impair Gag–Tsg101 interaction somewhat less efficiently than Hrs–Tsg101 interaction. Previous findings have shown that the MA domain appears not to be absolutely necessary for viral budding.^42, 43^ However, Dong *et al.*^44^ have shown that Gag-MA domain interacts with the adaptor complex AP3 which is involved in Gag trafficking. Ablation of AP3 function impairs HIV particle assembly by preventing Gag trafficking to multivesicular bodies. Thus, flotillin-1 is not the only endosomal trafficking factor that interacts with the Gag-MA domain.

We here show that flotillin-1 depletion surprisingly increases the formation of virus-like particles from Gag-transfected cells by about twofold both in 293T and HeLa cells. Although this increase is relatively modest, it is significant and may reflect the fact that the absence of flotillin-1 does not completely abrogate Gag–Tsg101 interaction. The mechanism of the increased release of VLPs upon flotillin-1 depletion is not known. As Tsg101 function is partially compromised, one would intuitively expect to see a decrease in VLP release. However, the phenotype and degree of change in viral release are similar to those observed upon depletion of the GGA proteins that are involved in endosomal sorting.^45^ Furthermore, it has been shown that cholesterol extraction and impairment of raft function results in increased release of viruses that are not capable of interacting with Tsg101.^46^ These conditions may be analogous to flotillin-1 ablation, which reduces Tsg101–Gag interaction due to removal of an important raft component, flotillin-1. The relatively modest degree of increase in VLP release upon flotillin-1 ablation may even be caused by a mixed effect, where a partial impairment of Tag101 function reduces VLP increase, but is compensated by an increase in VLP release caused by impairment of other functional aspects of flotillin-1 function, such as membrane rafts. This would result in the observed net effect: a modest but consistent increase in VLP release. However, further studies will be required to characterise the exact molecular reason for the increased VLP release upon flotillin depletion.

What is the molecular mechanism of flotillin-1 function during endosomal sorting of ubiquitinated cargo? Flotillins have been suggested to function as scaffolders for lipid microdomains, and they could thus affect sorting by clustering the cargo and the sorting proteins together. Such a clustering function for flotillins in the plasma membrane has been suggested by several publications for various cargo proteins.^19, 47, 48^ Intriguingly, Balbis *et al.*^49^ could show that highly ubiquitinated and phosphorylated EGFR accumulates in flotillin-1-enriched detergent-resistant membranes isolated from rat liver, providing first implications for a role for flotillin-1 microdomains in the endosomal sorting of EGFR.

During endosomal sorting, ubiquitinated cargo is clustered into microdomains that are covered by a clathrin coat ^50^ and contain Hrs,^7, 25^ but it is not known what the driving force for cargo clustering is. Interestingly, knockdown of CHC results in endosomal accumulation of flotillins,^51^ suggesting that there is a functional connection between flotillins and clathrin. In addition, deletion of the clathrin-binding motif in the C-terminus of Hrs largely abrogated flotillin-1 binding, although the interaction between Hrs and flotillin-1 is a direct one and not mediated by clathrin. Consistently, we here show that Hrs and CHC containing endosomal domains are still present in flotillin-ablated cells, speaking against a passive domain scaffolding function of flotillins in endosomal cargo sorting. A more active sorting function in endosomes is also supported by the fact that flotillins interact with both the cargo (e.g. EGFR, BACE1 and HIV-Gag)^15, 19^ and the sorting proteins, including Hrs and Tsg101.

**Figure 8:**
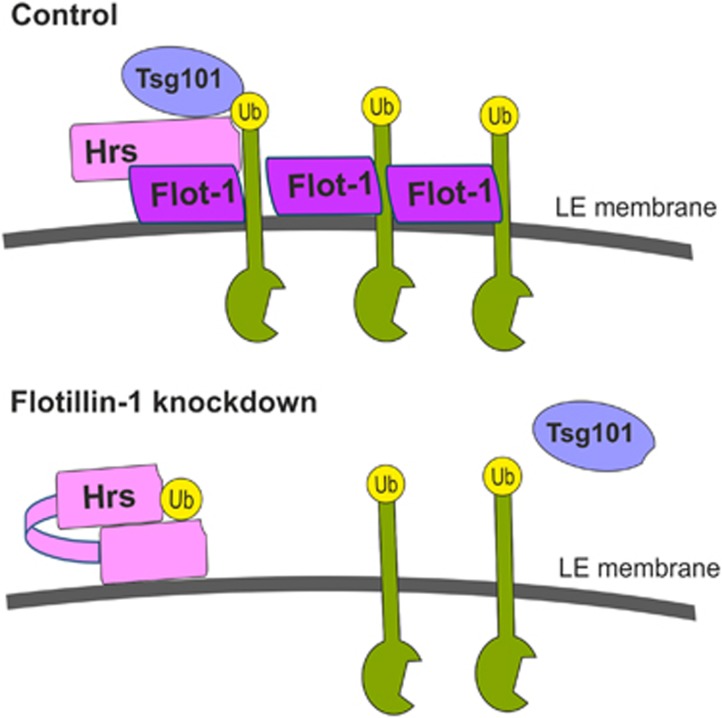
Model for the function of flotillin-1 in endosomal sorting. The model shows the events that take place in the endosomal membrane after EGF stimulation. In control cells (upper part), flotillin-1 is required for Hrs to obtain a conformation that is compatible with the binding of cargo ubiquitin and Tsg101/ESCRT-I. Hereby, flotillin-1 interacts with both the cargo and Hrs. Upon flotillin-1 depletion (lower part), Hrs is capable of interacting with neither the cargo nor Tsg101, although it resides on the endosomal membrane. This may suggest that flotillin-1 is involved in the release of the autoinhibited conformation of Hrs that results from monoubiquitination upon EGF stimulation.

Another possibility, which is also better supported by our data, how flotillin-1 might function in cargo transfer between the ESCRT complexes is the regulation of their ubiquitin-binding capacities. In Figure 8, we have provided a cartoon model for this putative function of flotillin-1 in ESCRT-mediated sorting. We have here shown that flotillin-1 is required for Hrs to bind ubiquitin (and thus cargo) upon EGF stimulation, but not in starved cells (see Figure 5e). Upon EGF stimulation, Hrs is monoubiquitinated and can be autoinhibited by the binding of its UIM domain to the internal ubiquitin.^52^ It is possible that flotillin-1 is required for the opening of this autoinhibited Hrs conformation on the endosomal membrane, but this possibility still needs to be experimentally addressed.

In addition to the cargo ubiquitin interaction, Hrs and HIV-1 Gag interaction with Tsg101 also requires flotillin-1. Both Hrs and Tsg101 are regulated by the binding of their UIM (Hrs) or UEV (Tsg101) domains to PTAP/PSAP type of motifs.^12, 13^ Hrs contains a PSAP motif that can interact with the Tsg101 UEV domain. On the other hand, the PTAP motif in Tsg101 can also interact with Tsg101 UEV domain and impair Tsg101 association with Hrs. The interaction regions in Hrs for Tsg101 (PSAP, C-terminal part including coiled-coil/GAT domain^13^) and flotillin-1 (VHS domain, PSAP, C-terminal part, especially the clathrin-binding motif) are partially overlapping, and more than one Hrs region appears to be necessary for a full interaction. However, flotillin-1 and Tsg101 do not compete for Hrs binding, as depletion of flotillin-1 impairs Hrs–Tsg101 interaction. It has been suggested that the opening of the autoinhibited conformation of Hrs or Tsg101 might be accomplished by an increased density of ubiquitin in the membranes by means of cargo clustering, which may involve flotillin-1. However, it still remains to be directly addressed how the cargo transfer from Hrs to Tsg101 is accomplished. Our data would be consistent with the model in which flotillin-1 is required for Hrs to obtain its capacity to bind ubiquitinated cargo and ESCRT-I (Figure 8), but the exact molecular details still need to be addressed in further experiments.

Taken together, our above data reveal flotillin-1 as a novel factor that assists ESCRT-mediated sorting of ubiquitinated cargo proteins towards lysosomes. As degradation of EGFR is essential for adequate regulation of proliferative signals, our findings are also of significance for human hyperproliferative diseases, especially cancers. Recent findings have linked flotillin overexpression with various types of cancers, and our data show that flotillin-1 depletion in breast cancer cells may result in overexpression of the EGFR.^53^ Therefore, flotillins evidently regulate the proliferative signalling axis by several means, including receptor activation, degradation and downstream signalling.

## Materials and methods

### Antibodies and constructs

Rabbit polyclonal antibodies against flotillin-1 (F-1180; IP: 1:300) and -2 (F-1680; IF: 1:100, IP: 1:300) were purchased from Sigma-Aldrich (Taufkirchen, Germany). Mouse monoclonal antibodies against GGA3 (cat. no. 612311; WB: 1:1000), flotillin-1 (cat. no. 610820, clone 18; IF: 1:100, WB: 1:1000) and flotillin-2 (cat. no. 610383; IF: 1:100, WB: 1:1000) were obtained from Transduction Labs (Franklin Lakes, NJ, USA). Mouse monoclonal antibody against Hrs (sc-271455, clone C-7; WB: 1:1000), CD63/LAMP3 (sc-5275; IF: 1:200), ubiquitin (sc-8017, clone P4D1; WB: 1:1000, IF: 1:100) and EGFR (sc-120, clone 528; IF: 1:100, WB: 1:1000) as well as rabbit polyclonal antibodies against Hrs (sc-30221, clone M-79; IF: 1:40, WB: 1:1000; IP: 1:100), Rab5 (sc-46692; IF: 1:200) and c-myc (sc-789; IP: 1:300) were from Santa Cruz Biotechnology (Santa Cruz, CA, USA). Mouse monoclonal antibodies against glyceraldehyde 3-phosphate dehydrogenase (GAPDH) (Ab8245; WB: 1:10 000), clathrin heavy chain (Ab2731; IF: 1:200) and Tsg101 (Ab83, clone 4A10; WB: 1:1000) and the rabbit polyclonal LAMP1 (Ab24170; IF: 1:500) antibody were obtained from Abcam (Cambridge, UK), and the mouse monoclonal anti-GFP (cat. no. 11814460001; WB: 1:1000) from Roche (Mannheim, Germany). The rabbit polyclonal anti-GFP (cat. no. 632592; IP: 1/300) was from Takara Bio (Mountain View, CA, USA). For immunofluorescence, primary antibodies were detected with Cy3- or Cy5-conjugated goat anti-mouse or anti-rabbit antibodies (Jackson Immunoresearch, West Grove, PA, USA) or with Alexa Fluor 488, 546 or 647 donkey anti-rabbit or anti-mouse antibodies (Molecular Probes, Karlsruhe, Germany). Rat flotillin-1 and -2 coding regions were cloned into pET-41a vector for bacterial expression as GST fusions (Merck/Millipore, Darmstadt, Germany). Human Tsg101, HIV-1 Gag and mouse Hrs as well as their subdomains used in this study were cloned into pGEX4T-1 or pEGFP vectors using PCR amplification. Gag-EGFP construct was obtained from Perlman and Resh,^54^ and the LTAL variant was produced by site-directed mutagenesis. The Hrs-EGFP constructs have been described previously.^52^ Tetraubiquitin-GST was obtained from Caixia Guo *et al.*^55^ TfnR-Ub^7^ was a kind gift from Harald Stenmark. Canine Rab5 carrying the Q79L mutation was PCR amplified from vector pGEM (a kind gift from Ivan Dikic) and cloned into pEGFP-C1 (Clontech, Mountain View, CA, USA). All DNA constructs were verified by sequencing.

### Cell culture, siRNA knockdown and growth factor treatment

Human cervix adenocarcinoma HeLa cells (ATCC; CCL-2; Manassas, VA, USA) and 293T cells (ATCC; CRL-3216) were grown in Dulbecco’s modified Eagle’s Medium (DMEM; Life Technologies, Darmstadt, Germany), with high glucose, supplemented with 10% foetal calf serum (Life Technologies), 100 U/ml penicillin and 100 μg/ml streptomycin (both Life Technologies) at 37 °C and 8% CO_2_. The cells were regularly tested for mycoplasma contamination by a PCR-based assay. Jurkat cells (clone E6.1; ATCC; TIB-152) were maintained at 37 °C and 5% CO_2_ in suspension in RPMI medium supplemented with 10% foetal calf serum, 100 U/ml penicillin and 100 μg/ml streptomycin. A transient knockdown of flotillins in HeLa cells was performed as described earlier^19, 51, 56^ using the Stealth siRNA System (Life Technologies). GGA3 siRNAs were purchased from Life Technologies. For control transfections, an oligo that does not target any human sequence (Stealth RNAi Negative Control, medium GC content) was used. Experiments were performed 72 h after transfection. For growth factor stimulation, the cells were kept under serum-free conditions for at least 18 h before the experiment. Stimulation with EGF was performed with a final concentration of 100 ng/ml and 37 °C for the indicated time points.

### CRISPR/Cas9 knockout of flotillin-1 in HeLa and 293T cells

The guide RNAs cloned into vector pD1301-AD (DNA2.0, Newark, CA, USA) were kindly provided by the Horizon Discovery Project (Cambrige, UK). The guide RNA plasmid against the sequence 5′-CTCCAGCCACCATGACTGGG-3′ was transiently transfected into HeLa cells to knock out flotillin-1 expression. Single-cell clones were selected that show no residual flotillin-1 expression. The clones were checked for the presence of the endosomal defects by LAMP3 staining.

### Immunofluorescence analysis

Cells grown on coverslips, either stimulated with EGF for the indicated time points or grown under regular growth conditions, were either fixed for 20 min at room temperature in 4% paraformaldehyde (80 mM PIPES (pH 6.8), 2 mM MgCl_2_, 5 mM EGTA (pH 8.0), 4% PFA) and permeabilised with 50 μg/ml digitonin, or fixed and permeabilised in cold methanol for 10 min at −20 °C. Fixed cells were labelled with primary antibodies and Cy3-, Cy5- or Alexa Fluor-conjugated secondary antibodies, and the nuclei were stained with 1 μg/ml 4′,6′-diamidino-2-phenylindole (DAPI). The cells were embedded in Gelmount (Biomeda, Foster City, CA, USA) supplemented with 50 mg/ml 1,4-diazadicyclo(2,2,2)octane (Fluka, Neu-Ulm, Germany). Specimens were examined using a confocal laser scanning microscope (Zeiss LSM 710 combined with Axio Observer, Carl Zeiss, Jena, Germany).

### Treatment with fluorophore-labelled reagents

For the visualisation of lysosomes, the fluorescent dye Lysotracker (Life Technologies) was diluted 1:5000 in the medium and the cells were treated for 30 min at 37 °C. In cases where the cells were chased with Alexa Fluor 488-coupled EGF (Life Technologies), they were incubated with the fluorescent EGF (dilution 1:500, 100 ng/ml) for 30 min on ice. Subsequently, the cells were washed with warm DMEM without growth factors and placed to 37 °C for 30 min to allow the fluorescent EGF to be internalised. The transferrin uptake for experiments with the transferrin receptor-ubiquitin chimera was performed as described previously^7^ with minor modifications. In brief, starved HeLa cells were incubated with 50 μg/ml Alexa Fluor 546-Tfn for 15 min at 37 °C. Subsequently, the cells were washed two times in DMEM and chased for 60 min in DMEM containing 10 μg/ml cycloheximide and 0.3 mM leupeptin at 37 °C. Cells were fixed in 4% PFA and processed as described above.

### Cell lysis for western blotting

Cells were lysed in lysis buffer (50 mM Tris (pH 7.4), 0.15 m NaCl, 2 mM EDTA, 1% NP-40) supplemented with proteinase inhibitor cocktail (Sigma-Aldrich) for 30 min on ice. Cellular debris were pelleted for 12 min at 20 000 *g*, and protein concentrations of the supernatants were determined with Bio-Rad protein assay (Bio-Rad, Munich, Germany) and equal protein amounts were analysed by 10% sodium dodecyl sulphate–polyacrylamide gel electrophoresis (SDS–PAGE) and western blot.

### Co-immunoprecipitation

For co-immunoprecipitation experiments, the cells were lysed in co-immunoprecipitation buffer (10 mM Tris (pH 8.0), 0.15 m NaCl, 5 mM EDTA, 0.5% Triton X-100, 1 mM sodium orthovanadate, 5 mM NaF, 60 mM *n*-octyl-β-d-glucopyranoside) supplemented with proteinase inhibitor cocktail (Sigma-Aldrich). Cellular debris were pelleted for 12 min at 20 000 *g* and removal of unspecific binding material from the supernatants was accomplished by preclearing with 50 μl of Pansorbin beads (Calbiochem, Darmstadt, Germany). Equal protein amounts of the lysates were incubated overnight at 4 °C with 30 μl of magnetic Dynabeads Protein A (Life Technologies) precoupled with rabbit anti-Hrs or c-myc (Santa Cruz Biotechnology) or with rabbit anti-GFP (Takara Bio). Beads were then washed four times with 1 ml co-immunoprecipitation buffer, heated for 5 min at 94 °C in 20 μl SDS–PAGE loading buffer supplemented with 5% β-mercaptoethanol and 25 mM dithiothreitol and subjected to SDS–PAGE and western blotting.

### Expression and purification of recombinant proteins

Bacterial expression vectors encoding rat flotillin-1-GST and flotillin-2-GST have been described before.^57^ Human Tsg101-GST, mouse Hrs-FL-GST, Hrs-ΔUIM-GST, Hrs-LSAL-GST, Hrs-ΔVHS-GST, VHS-GST, Hrs-405-GST, Hrs-560-GST, HIV-1 Gag-FL-GST, Gag-MA-GST, Gag-CA-GST, Gag-p6-GST and dMA-Gag-GST were cloned using PCR amplification from existing constructs,^52, 54^ using standard techniques. The PTAP motif was modified to LTAL by site-directed mutagenesis. The constructs were transformed into the bacterial expression strain *E. coli* Rosetta (De3)pLysS (Millipore), grown in a liquid culture at 37 °C to OD_600_ 0.4–0.6. Transformants were either induced with 0.15 mM isopropyl β-d-1-thiogalactopyranoside overnight at 19 °C or for 4–6 h at 37 °C with 1 mM isopropyl β-d-1-thiogalactopyranoside. The bacterial pellet was washed once in phosphate-buffered saline (PBS) and lysed in GST lysis buffer (50 mM HEPES (pH 7.5), 0.15 m NaCl, 1 mM EDTA, 5% glycerol, 0.1% Nonidet P-40) supplemented with 100 μg/ml lysozyme, 1 mM dithiothreitol and proteinase inhibitors. Recombinant GST proteins were immobilised on glutathione-sepharose beads (GE Healthcare, Munich, Germany).

### GST-pulldown assays

HeLa cells were lysed in co-immunoprecipitation buffer supplemented with proteinase inhibitors. Lysates were incubated 3 h or overnight at 4 °C with purified GST-tagged recombinant proteins (5–20 μg) immobilised on glutathione-sepharose beads. Beads were washed four times with co-immunoprecipitation buffer and cooked in reducing SDS–PAGE loading buffer. For the analysis of a direct interaction between Hrs and flotillins, 1 U of thrombin was used to cleave the GST-tag from 20 μg recombinant full-length Hrs-GST. Flotillin-1-GST was cleaved for the rescue experiments of Hrs/ubiquitin binding. Cleavage was performed in cleavage buffer (20 mM Tris-HCl (pH 8.4), 150 mM NaCl, 2.5 mM CaCl_2_, 1 mM dithiothreitol) overnight at 19 °C. After inactivation of thrombin with 1 mM phenylmethylsulfonyl fluoride, the cleaved protein was added to the respective pulldown. For the direct interaction, cleaved Hrs was added to 5 μg of GST (pET-41a), flotillin-1-GST or flotillin-2-GST in 50 mM Tri-HCl (pH 7.5), 150 mM NaCl, 1 mM EDTA, 1 mM dithiothreitol and 0.01% Triton X-100 for 3 h on ice. After washing the beads three times in the aforementioned buffer, the samples were cooked in 2x SDS–PAGE loading buffer and analysed by western blot.

### Ferrofluid-based isolation of endosomes

A similar method has been described before.^58^ For the coupling of EGF or BSA to Ferrobeads (FerroTec, Bedford, NH, USA), 50 μl of Ferrobeads were diluted in 1.5 ml PBS and either coupled with 30 μg human recombinant EGF (Sigma-Aldrich) or 300 μg protease-free BSA (PAA Laboratories, Linz, Austria) by shaking overnight at 4 °C. For reconstitution of coated Ferrobeads, a MiniMACS Separator Column (Miltenyi Biotec, Bergisch Gladbach, Germany) attached to a magnetic holding device was first equilibrated with 500 μl PBS and then loaded with the Ferrobead complexes. The column was washed with 1.2 ml PBS and the Fe-EGF/-BSA were eluted with 2 ml PBS after removing the column from the magnetic standing device. From the resulting Fe-EGF and Fe-BSA solutions, 200 μl per 10 cm dish were used to stimulate cells for 30 min at 37 °C. After internalisation, cells were washed with PBS, pelleted and resuspended in 500 μl endosome buffer (10 mM HEPES (pH 7.2), 100 mM KCl, 1 mM EDTA, 25 mM sucrose) supplemented with protease inhibitors, 5 mM NaF and 1 mM sodium orthovanadate. Homogenisation of the cells was performed by passing them 2x20 times through a 23 G needle with a 10 min centrifugation step at 1000 *g* in between. Supernatants were collected and input sample prepared for SDS–PAGE. Equilibrated MiniMACS Separator Columns were loaded with the supernatants. After thorough washing of the columns with 5 ml endosome buffer each, isolated endosomes were eluted after removing columns from magnetic standing device with 200 μl endosome buffer supplemented with additional 60 mM N-octylglucoside. Isolated endosomes were analysed by SDS–PAGE.

### Ground-state depletion microscopy

Control cells and cells depleted of flotillin-1 were stimulated with 100 ng/ml EGF, fixed in methanol and blocked in 1% BSA/PBS. Cells were immunostained for LAMP3 and a secondary Alexa Fluor 647-coupled antibody. For super-resolution microscopy, a Leica SR GSD 3D (Leica Microsystems, Wetzlar, Germany) with an HC PL APO x160 objective (Leica Microsystems) was used. Samples were embedded in Vectashield/Glycerol-Tris-buffer (Vectashield and Glycerol-Tris-buffer in a 1:10 ratio; glycerol with 50 mM Tris (pH 8)). Cells were analysed using the Leica LAS AF Software (Leica Miicrosystems, Wetzlar, Germany) and GraphPad Prism.

### Electron microscopy

Cells grown on 10 cm dishes were chased with Ferro-EGF for 2 h, fixed with 1.5% glutaraldehyde and 1.5% formaldehyde in 0.15 m HEPES buffer (pH 7.4) for 30 min at room temperature, scraped and pelleted. The cell pellets were postfixed for 60 min with 1% osmium tetroxide in 50 mM HEPES buffer (pH 7.5). After washing, the samples were incubated overnight in a 2% aqueous uranyl acetate solution. The next day, the cells were dehydrated in a graded series of ethanol, embedded in Agar100 and polymerised at 60 °C for 24 h. Ultrathin sections (60–90 nm) of the embedded pellets were cut with a Reichert/Leica Ultracut E microtome (Leica Microsystems). Sections were analysed with a Zeiss EM 902 transmission electron microscope (Carl Zeiss, Oberkochen, Germany) at 80 kV. Electron micrographs were recorded using a slow-scan 2 K CCD camera (TRS; Tröndle, Moorenweis, Germany).

### Virus-like particle measurement

The 293T cells (control and flotillin-1 knockout) were transfected with Gag-EGFP, and the cells were transferred onto 10 cm plates in 8 ml 2% foetal calf serum/DMEM. After 48 h, the medium and cells were collected and the pellet was lysed in 2 ml of lysis buffer. One millilitre of the medium was filtered through a 0.2 μm filter and centrifuged through a 400 μl sucrose cushion (20% in PBS) for 90 min 20 000 *g*. VLPs were collected, lysed in 130 μl of lysis buffer supplemented with gel loading buffer. An aliquot of the lysate and VLP fraction were analysed by western blot and quantified. HeLa cells were transiently transfected with Gag-EGFP and siRNAs against flotillin-1. The cells were transferred onto 10 cm plates. At ~70% confluency, the medium was exchanged and the cells were incubated for 48 to 72 h to allow VLP secretion. The collected media were centrifugated at 5000 r.p.m. for 3 min to remove cell debris, and again at 20 000 *g* for 15 min. Cell pellets were lysed in 2 ml of lysis buffer. An aliquot of the clarified cell lysate and the medium was used for the measurement of p24 by an ELISA Kit (R&D Systems, Wiesbaden, Germany). The percentage of VLPs released into the medium (% total) was calculated.

### Statistical analysis

Unless otherwise stated, experiments were performed at least three times. Densitometric analysis was accomplished with Quantity One Software (Bio-Rad, Munich, Germany). GraphPad Prism Software (Version 5; GraphPad Software, La Jolla, CA, USA) was used to statistically analyse the data. Student’s *t*-test (two-tailed, unpaired), one- or two-way ANOVA were applied, as appropriate. Data are presented as mean±s.d. Significance is shown versus the respective control and depicted as follows: *P<0.05, **P<0.01 and ***P<0.001.

### Data availability

All relevant data and detailed protocols are available from the authors upon request.
