# Supplementary material for: Regulation of cargo transfer between ESCRT-0 and ESCRT-I complexes by flotillin-1 during endosomal sorting of ubiquitinated cargo
Source: Oncogenesis. 2017 Jun 5;6(6):e344–. doi: 10.1038/oncsis.2017.47 (PMC5519196; doi:10.1038/oncsis.2017.47)
Supplement: Supplementary Figure S1 [file oncsis201747x1.pdf]

Supplementary Figure S1, Meister et al.

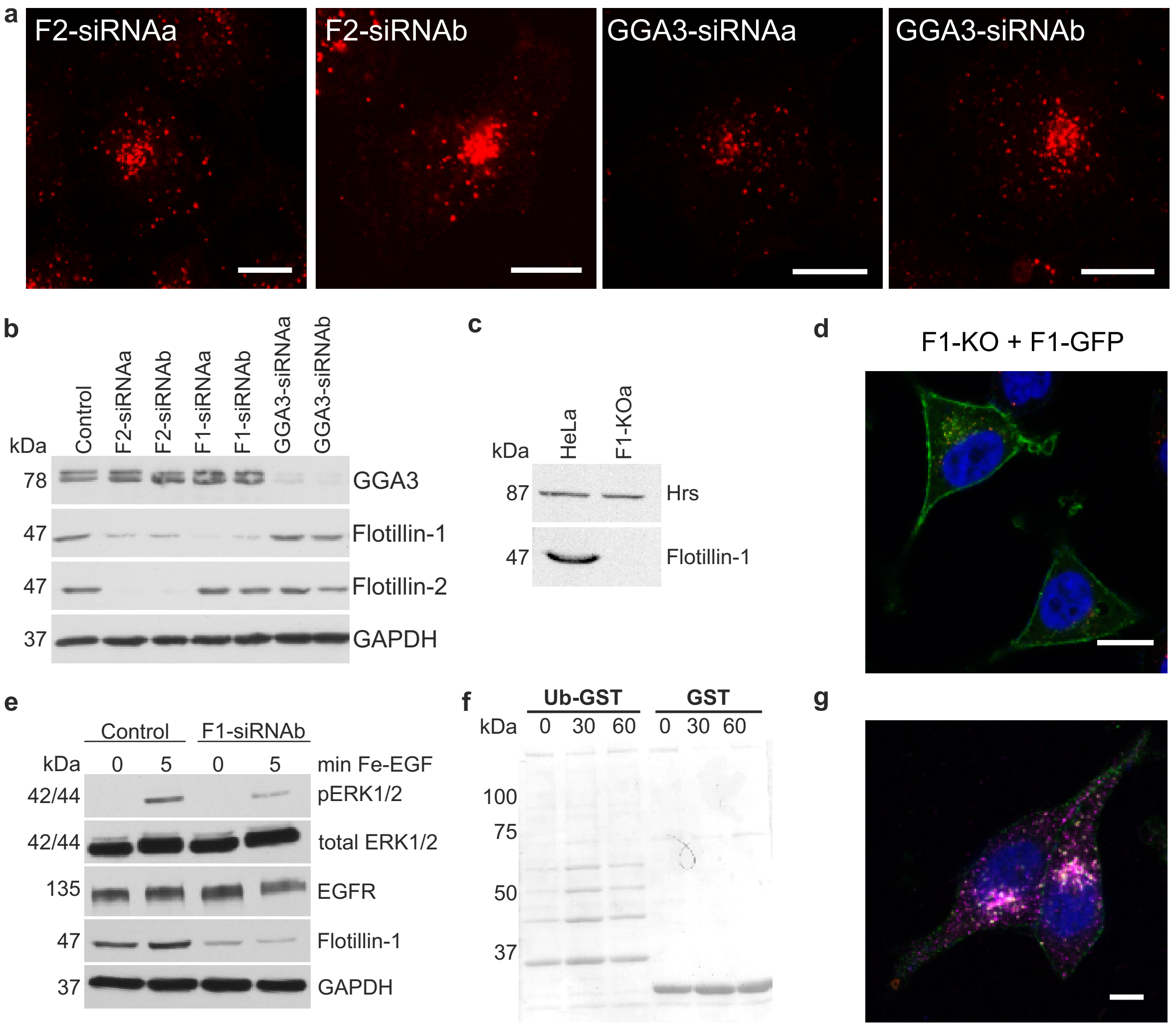

Supplementary Figure S1: EGFR degradation is impaired in the absence of flotillins.
(a) Flotillin-2 or GGA3 knockdown cells were starved overnight and stimulated with 100 ng/ml EGF for 60 min, fixed and immunostained for EGFR. (b) Knockdown efficiencies for flotillin-1, flotillin-2 and GGA3 for experiments shown in figures 1 and S1a. (c) Absence of flotillin-1 expression in flotillin-1 CRISPR/Cas9 knockout cells. (d) Expression of flotillin-1-EGFP in cells shown in Figure 1c. (e) Control and flotillin-1 knockdown cells were starved and incubated with Ferrofluid-coupled EGF for 5 min. The activation of extracellularly-regulated kinase ERK was studied with phospho-specific antibodies against phosphorylated ERK (pERK) by Western blot. (f) Ponceau staining shows the amount of GST fusion proteins for the ubiquitin-GST pulldown shown in figure 2d. (g) Expression of flotillin-1-EGFP in cells shown in Figure 3e. Scale bar in a, d, g: 10 µm.
