# Supplementary material for: Regulation of cargo transfer between ESCRT-0 and ESCRT-I complexes by flotillin-1 during endosomal sorting of ubiquitinated cargo
Source: Oncogenesis. 2017 Jun 5;6(6):e344–. doi: 10.1038/oncsis.2017.47 (PMC5519196; doi:10.1038/oncsis.2017.47)
Supplement: Supplementary Figure S2 [file oncsis201747x2.pdf]

Supplementary Figure S2, Meister et al.

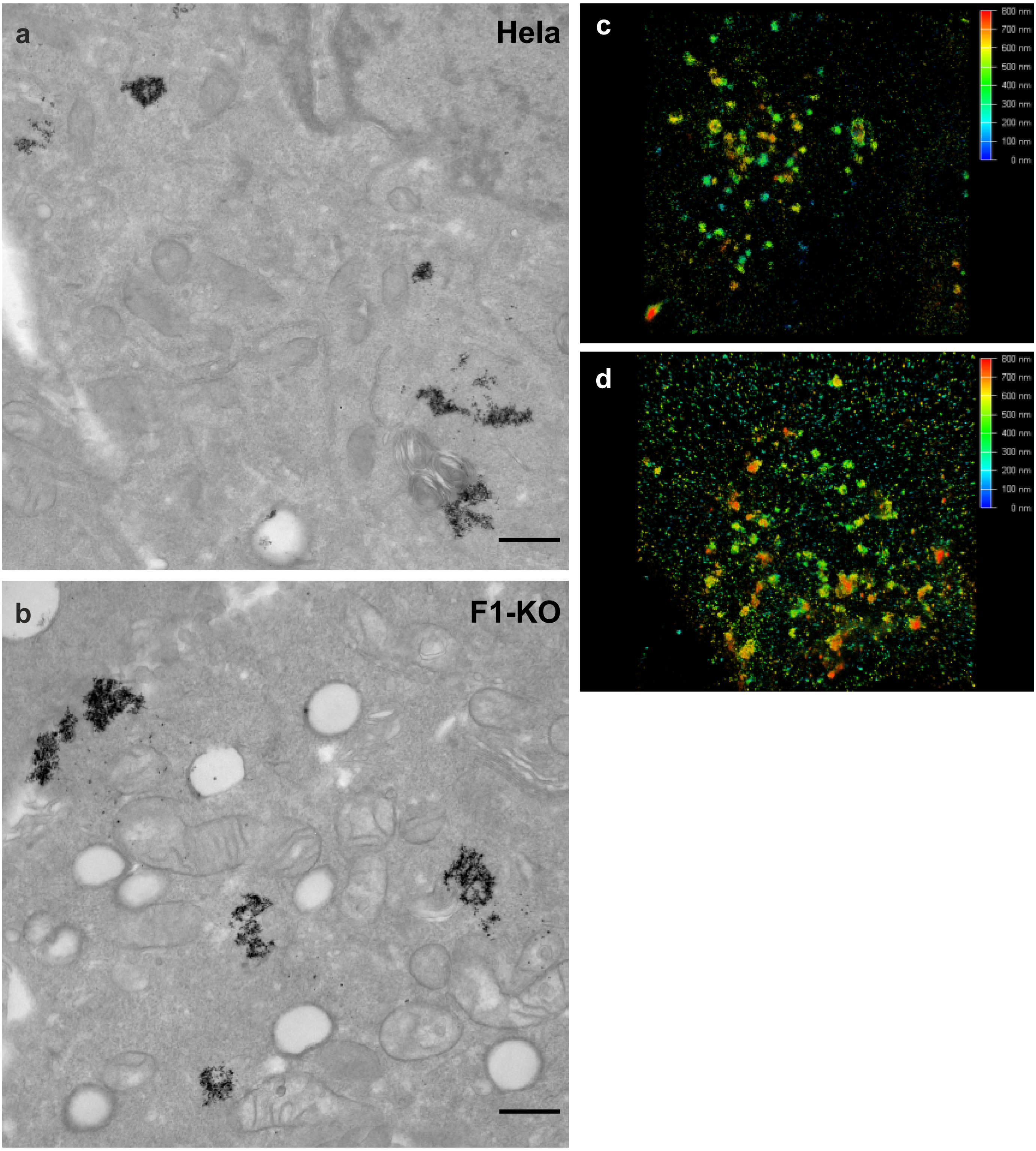

Supplementary Figure S2: Enlarged late endosomes in flotillin-1 ablated cells.
(a-b) Larger images of the EM micrographs shown in Fig. 4a. **(c)** Control or **(d)** flotillin-1 knockdown HeLa cells were immunostained for LAMP3 and imaged by 3-dimensional ground-state depletion microscopy with optical z sections. Color coding shows the position of the respective structure in a 3D-overlay of the sections.
