# Supplementary material for: Regulation of cargo transfer between ESCRT-0 and ESCRT-I complexes by flotillin-1 during endosomal sorting of ubiquitinated cargo
Source: Oncogenesis. 2017 Jun 5;6(6):e344–. doi: 10.1038/oncsis.2017.47 (PMC5519196; doi:10.1038/oncsis.2017.47)
Supplement: Supplementary Figure S3 [file oncsis201747x3.pdf]

Supplementary Figure S3, Meister et al.

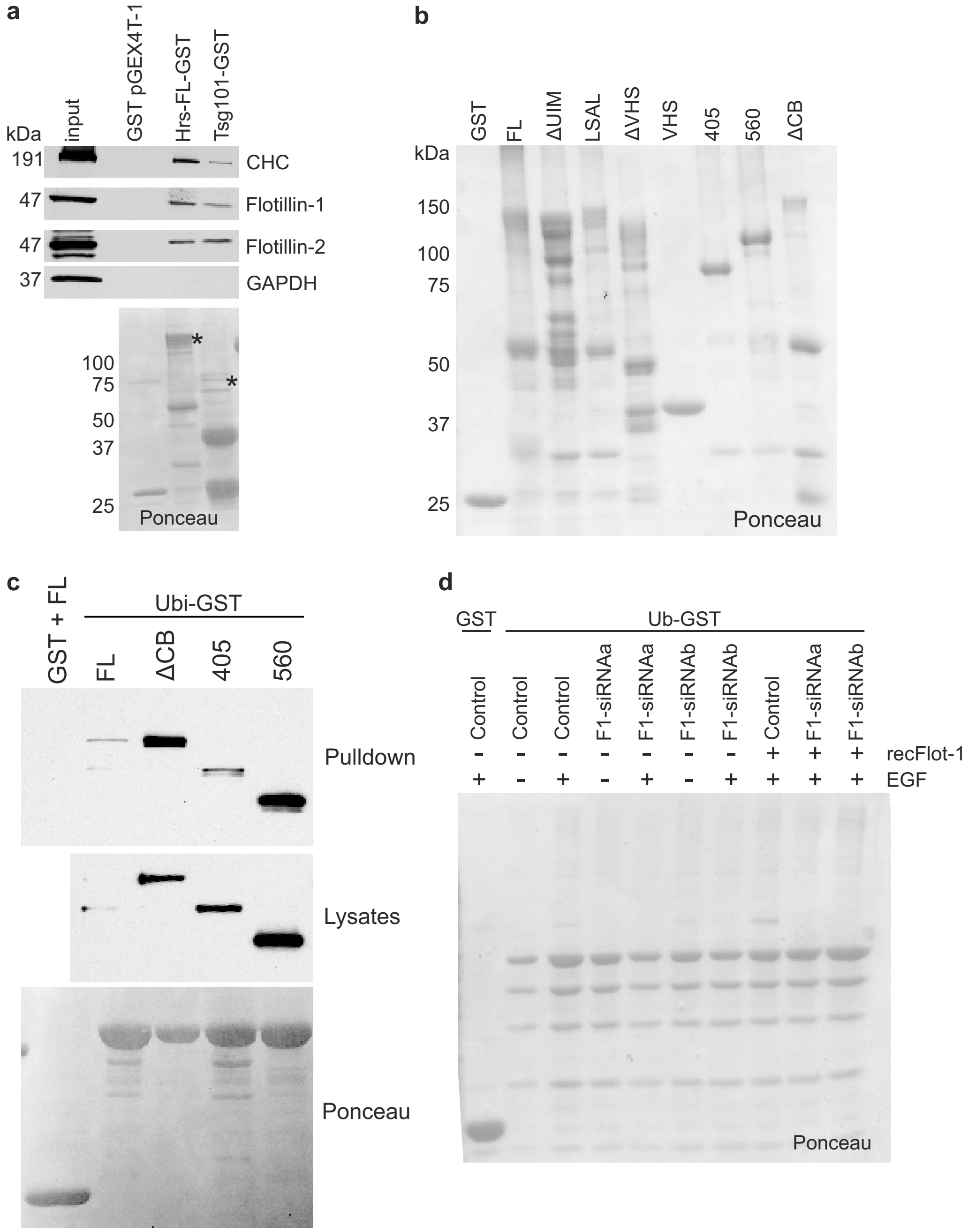

Supplementary Figure S3. Flotillins interact with Hrs and Tsg101.
(a) GST pulldown was performed from HeLa cell lysates using purified full-length Hrs-GST and Tsg-101-GST. Bound proteins (CHC, flotillin-1 and flotillin-2) were detected with specific antibodies, GAPDH was used as a negative control. **(b and d)** Ponceau staining of the blots shown in **(b)** Figure 5b, and **(d)** Figure 5e shows the amount of GST fusion proteins used in the said experiments. **(c)** Ubi-GST-pulldown of EGFP-tagged Hrs deletion mutants expressed in HeLa cells.
