# Supplementary material for: Regulation of cargo transfer between ESCRT-0 and ESCRT-I complexes by flotillin-1 during endosomal sorting of ubiquitinated cargo
Source: Oncogenesis. 2017 Jun 5;6(6):e344–. doi: 10.1038/oncsis.2017.47 (PMC5519196; doi:10.1038/oncsis.2017.47)
Supplement: Supplementary Figure S4 [file oncsis201747x4.pdf]

Supplementary Figure S4, Meister et al.

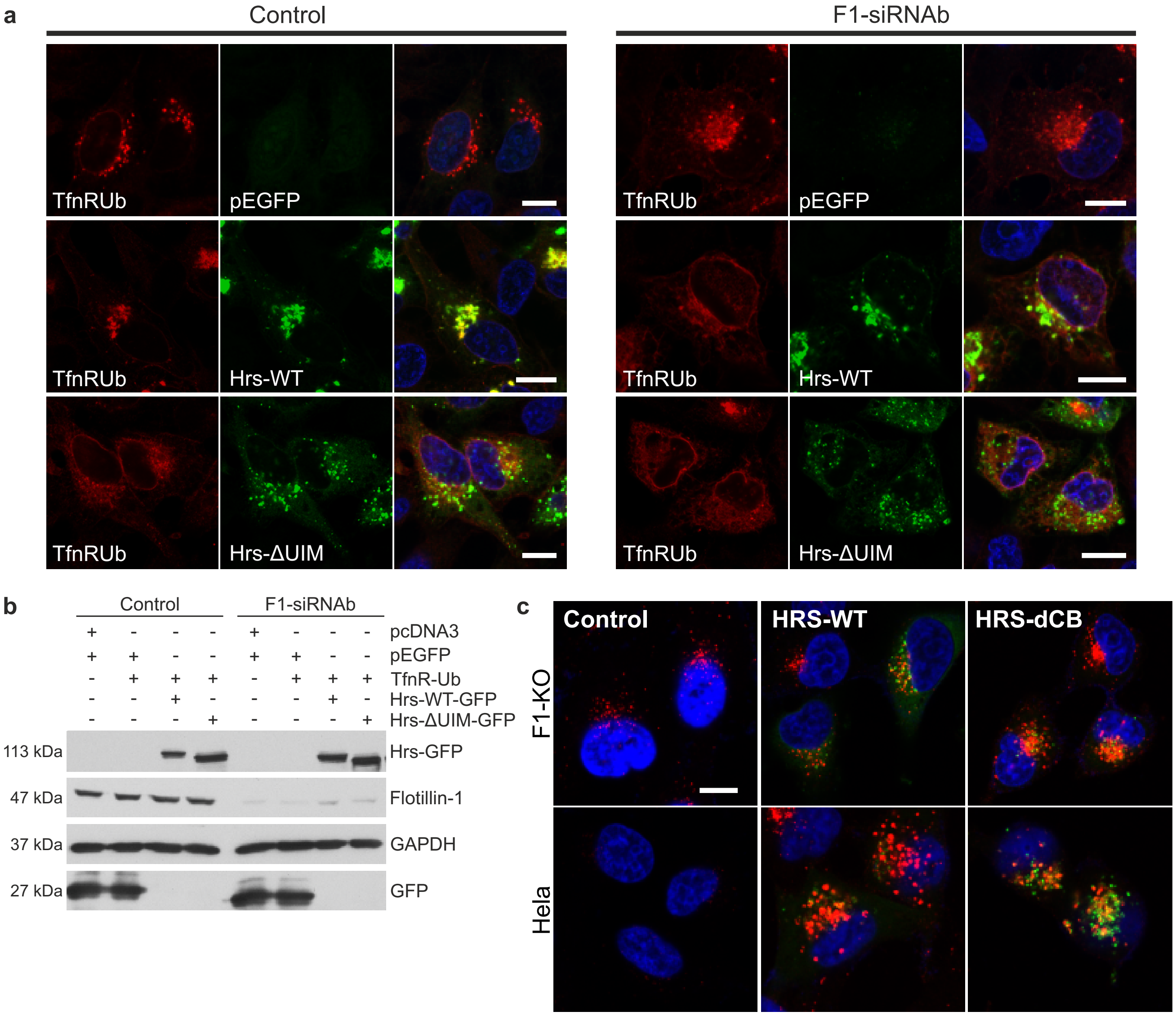

Supplementary Figure S4. Flotillin-1 is necessary for the sorting of ubiquitinated cargo by ESCRT-0. The expression of transfected proteins used in Figure 6 as detected by immunofluorescence. **(a)** Control and flotillin-1 knockdown HeLa cells were transfected with TfnR-Ub, Hrs-WT-GFP or Hrs-ΔUIM-GFP or pEGFR as control, fixed with methanol and immunostained for TfnR. Scale bar: 10 µm. **(b)** Knockdown efficiency (anti flotillin-1) and expression of GFP fusion proteins (anti-GFP) were monitored by Western blot. GAPDH was used as a control for equal loading. **(c)** HeLa cells or flotillin-1 knockout cells were transfected with the indicated HRS-EGFP constructs, starved, treated with EGF for 60 min and immunostained for EGFR. Red: EGFR, green: Hrs-EGFP, blue: DAPI.
