# Supplementary material for: Regulation of cargo transfer between ESCRT-0 and ESCRT-I complexes by flotillin-1 during endosomal sorting of ubiquitinated cargo
Source: Oncogenesis. 2017 Jun 5;6(6):e344–. doi: 10.1038/oncsis.2017.47 (PMC5519196; doi:10.1038/oncsis.2017.47)
Supplement: Supplementary Figure S5 [file oncsis201747x5.pdf]

Supplementary Figure S5, Meister et al.

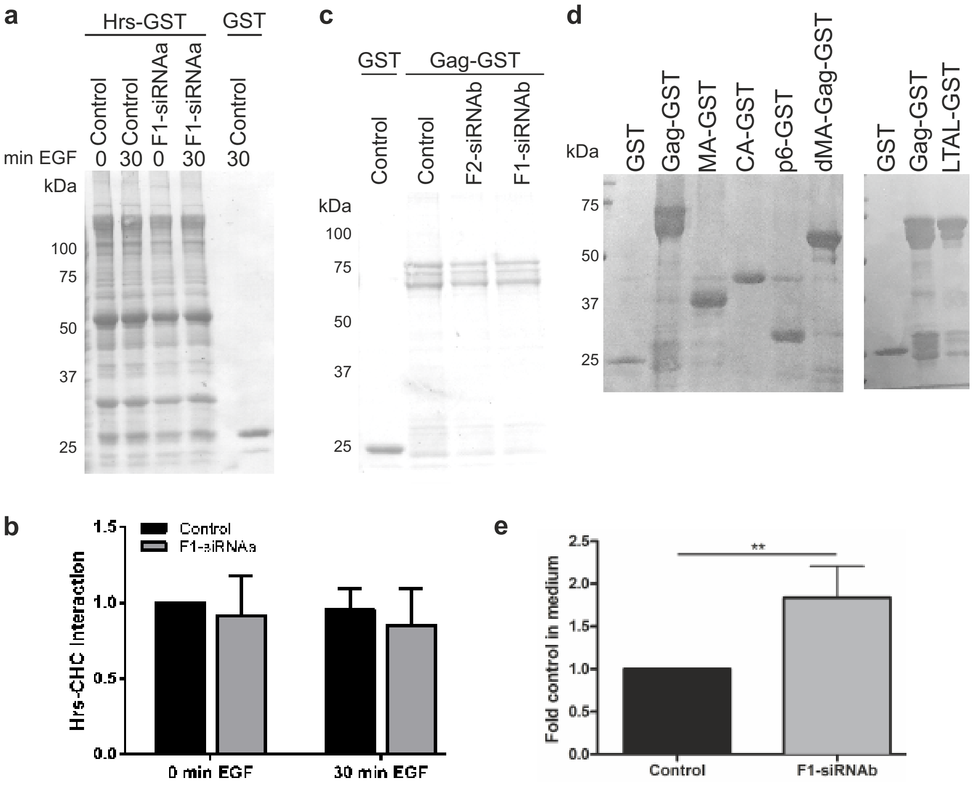

Supplementary Figure S5. Flotillins and HIV-1 Gag polyprotein.
Control of the amount of recombinant proteins used: Ponceau staining of blots shown in **(a)** Figure 7a, **(b)** figure 7c, and **(c)** figure 7e+f. **(d)** Flotillin-1 depletion does not significantly impair Hrs-GST binding to CHC. Two-way ANOVA with Bonferroni post-test, mean ± SD of 3 independent experiments. **(e)** VLPs from Gag-EGFP transfected control and flotillin-1 knockdown HeLa cells were collected, and the amount of Gag in the cell lysates and in the medium was determined by ELISA for p24 part of Gag. The percentage of released Gag was calculated, and the data are presented as fold Gag released in the medium as compared to control siRNA cells.
